# Case-control study of anthropometric measures and testicular cancer risk

**DOI:** 10.3389/fendo.2012.00144

**Published:** 2012-11-26

**Authors:** Fabrizio Giannandrea, Donatella Paoli, Francesco Lombardo, Andrea Lenzi, Loredana Gandini

**Affiliations:** Laboratory of Seminology – Semen Bank, Department of Experimental Medicine, University of Rome “La Sapienza”Rome, Italy

**Keywords:** testicular cancer, height, weight, body mass index, body size

## Abstract

The etiology of *testicular* germ cell tumors (TGCTs) is poorly understood. Recent epidemiological findings suggest that, TGCT risk is determined very early in life, although the available data are still conflicting. The rapid growth of the testes during puberty may be another period of vulnerability. Body size has received increasing attention as possible risk factor for TC. To clarify the relation of body size and its anthropometric variables to TGCT risk, the authors analyzed data from 272 cases and 382 controls with regard to *height* (cm), weight (Kg), and body mass index (BMI; kg/m^2^). Overall, participants in the highest quartile of height were more likely to be diagnosed with TGCTs than participants in the lowest quartile of height, OR 2.22 (95% confidence intervals (CI): 1.25–3.93; adjusted; *p*_trend_ = 0.033). Moreover, histological seminoma subgroup was significantly associated with tallness, very tall men (>182 cm) having a seminoma TGCT risk of OR = 2.44 (95% confidence intervals (CI): 1.19–4.97; adjusted; *p*_trend_ = 0.011). There was also a significant inverse association of TGCT with increasing BMI (*p*_trend_ = 0.001; age-adjusted analysis) and this association was equally present in both histological subgroups. These preliminary results indicate that testicular cancer (TC) is inversely associated with BMI and positively associated with height, in particular with seminoma subtype. Several studies have reported similar findings on body size. As adult *height* is largely determined by high-calorie intake in childhood and influenced by hormonal factors at puberty, increased attention to postnatal exposures in this interval may help elucidate the etiology of TGCTs.

## Introduction

Testicular cancer (TC) is the most common type of malignancy in men aged 15–40 years (Dieckmann and Pichlmeier, 2004). The incidence of TC has more than doubled worldwide over the last 40 years in several populations (Purdue et al., 2005; Bray et al., 2006). The reasons of this rise are not entirely clear (McGlynn and Cook, 2009).

Evidence of incidence peak of TC among young adults suggests that causal factors operate very early in life (Bray et al., 2006). One theory attributes it to the increase in endogenous estrogen levels during prenatal life and/or later exposures to various occupational and environmental estrogenic chemicals, termed endocrine-disrupting chemicals (EDCs) (Toppari et al., 1996; Sharpe, 2003; Cook et al., 2011; Giannandrea et al., 2011). According to this hypothesis, these exposures are also risk factors for other male reproductive disorder, such as cryptorchism and impaired sperm quality (Skakkebaek et al., 2001; Storgaard et al., 2006). Recently, it has been suggested that post-natal exposures might increase the risk of developing TC, and that secretion of sex hormones, which are involved in the growth and the development of the testicle, might be instrumental in TC progression (James, 2010; Trabert et al., 2011).

One possible risk factor that has received increasing attention is body size, which is determined by hereditary, nutritional, and hormonal factors (Dieckmann and Pichlmeier, 2002). Height, weight, and body mass index (BMI) have been examined in a number of studies, with mixed results (Lerro et al., 2010). Several studies have reported inverse associations of TC with BMI (Petridou et al., 1997; Bjørge et al., 2006; Lerro et al., 2010). A number of studies have reported that increased adult height may be a risk factor for TC, thereby suggesting that factors related to adult height may also be related to risk of these tumors (Richiardi et al., 2003; Dieckmann et al., 2008; Lerro et al., 2010). One explanation for the height association may be that better childhood nutrition increases both adult height and risk of TC. This hypothesis is supported by epidemiological observations of constantly increasing incidence of TC since the beginning of the twentieth century with the only major interruption in this trend occurring for men born during World War II or immediately thereafter, when food availability had been dramatically reduced (Moller, 1989; Aschim et al., 2005; Giannandrea, 2009).

To clarify the relation of anthropometric variables to TGCT risk and to one another, we analyzed data from 321 cases and 465 controls enrolled at our laboratory with regard to height (cm), weight (Kg) and body mass index (BMI; kg/m^2^).

## Materials and methods

Participants among 321 consecutive patients affected by TC and 465 controls were originally selected at the Laboratory of Seminology, Semen Bank, Department of Experimental Medicine, University of Rome “La Sapienza,” Rome, Italy. Controls were recruited among healthy men who underwent to seminal analyses in order to ascertain their fertility status before getting married. We excluded men with conditions possibly associated with impaired sperm quality, and/or with a history of neoplastic pathologies to provide the most suitable control group. Only men aged 20–55 years at diagnosis were eligible for the study. Inclusion was restricted to this age range as normal height growth is complete between 16 and 20 years, while there is a characteristic decline in height growth in adulthood. Some of the controls who underwent to seminal analyses were men affected by severe obesity according to WHO classification (WHO, 2000), and therefore, this group was excluded from both cases and controls in order to avoid the confounding effect of this condition on our study variables.

Only patients with tumors of germ cell origin were included in the analyses. We categorized the cases into the two major histopathologic subgroups: seminomas or non-seminomas, which include embryonal carcinomas, endodermal sinus tumors, teratomas, choriocarcinomas, or germ cell tumors of mixed morphology. Our study population was therefore finally restricted to a total of 272 cases and 382 controls, according to the selection criteria described above.

Statistical analyses were performed by use of a SPSS statistical package. All tests were two sided, with *p* < 0.05 defined as statistically significant. Logistic regression models were calculated to obtain odds ratios (OR) and 95% confidence intervals (CIs) with adjustment for the potentially confounding factors, height, weight, and age at diagnosis (Armitage et al., 2001). ORs, confidence limits, and *p* values were adjusted for age because of imbalance of age distribution between cases and controls.

Height, weight, and BMI were categorized into quartiles on the basis of the distribution among the entire study group. Categories were 20–29 years, 30–34 years, 35–39 years, and ≥40 years with respect *t* to age; ≤173 cm, 174–178 cm, 179–182 cm, and >182 with respect to height. BMI [weight (in kilograms) divided by height (in meters) squared] was calculated for each subject and categorized into slim (BMI <23.15 kg/m^2^), normal weight (BMI 23.16–24.97 kg/m^2^), overweight (BMI 24.98–27.4 kg/m^2^), and obese (BMI ≥27.4 kg/m^2^). Tests for trend were performed by use of the categorized variables.

## Results

The descriptive tabulation of anthropometric measures distribution of cases and controls is shown in Table 1. The mean ages of the non-seminoma cases was lower than the mean age of the seminoma cases (30.6 years, standard deviation (SD): 6.0 vs. 34.1 years, SD: 5.9; *p* < 0.05). It is well-acknowledged that non-seminoma peak at younger age than seminoma. Therefore, the risk of TC decrease with increasing age at diagnosis, with non-seminomas having a more significant inverse relationship with age (*p*_trend_ < 0.001) than seminomas (*p*_trend_ = 0.021) (Table 2). The relations of anthropometric variables and TC risk are shown in Table 2. In the multivariate analysis, BMI was inversely associated and height was positively associated with risk of TC (Table 2).

**Table 1:** **Descriptive statistical analysis of anthropometric measures (Mean ± SD)**.

	**Patients (*n*)**	**Age (years)**	**Height (cm)**	**BMI**	**Weight (Kg)**
Controls (C)	382	35.7 ± 7.1	177.6 ± 6.5	24.7 ± 2.0	78.1 ± 8.7
All TC (TC)	272	32.4 ± 6.2	177.8 ± 6.3	23.8 ± 2.3	75.5 ± 9.0
Seminoma (S)	138	34.1 ± 5.9	177.5 ± 6.1	24.0 ± 2.5	75.7 ± 9.4
Nonseminoma (NS)	134	30.6 ± 6.0	178.2 ± 6.5	23.6 ± 2.2	75.3 ± 8.6
TC vs. C		b	a	a	a
S vs. C		a	a	a	a
NS vs. C		b	a	a	a

a Not significant.

b p < 0.05.

**Table 2:** **Relation of age, height, weight, and body mass index (BMI) to testicular germ cell tumor risk by histology**.

	**Controls (n. 382)**		**All TC (n. 272)**				**Seminoma (n. 138)**				**Nonseminoma (n. 134)**			
**Variable**	**No**.	**%**	**No**.	**%**	**OR**	**95% CI**	**No**.	**%**	**OR**	**95% CI**	**No**.	**%**	**OR**	**95% CI**
**AGE (YEARS)**														
20–29	56	15.1	82	30.7	1.0	Referent	29	21.2	1.0	Referent	53	40.8	1.0	Referent
30–34	93	25.0	85	31.8	0.63	0.38, 1.06	42	30.7	0.59	0.32, 1.08	43	33.1	0.76	0.35, 1.66
35–39	129	34.7	70	26.2	0.33	0.20, 0.56	47	34.3	0.41	0.22, 0.77	23	17.7	0.25	0.12, 0.52
≥40	94	25.3	30	11.2	0.24	0.13, 0.56	19	13.9	0.41	0.21, 0.81	11	8.5	0.14	0.06, 0.30
					*p* trend < 0.001				*p* trend = 0.021				*p* trend < 0.001	
**HEIGHT (cm)**														
≤173	108	28.3	68	25.0	1.0	Referent	32	23.2	1.0	Referent	36	26.9	1.0	Referent
174–178	98	25.7	81	29.8	1.62	0.99, 2.66	50	36.2	1.88	0.99, 3.54	31	23.0	1.37	0.73, 2.54
179–182	93	24.3	54	19.9	1.37	0.83, 2.25	23	16.7	1.03	0.61, 1.98	31	23.0	1.77	0.92, 3.40
>182	83	21.7	69	25.4	2.22	1.25, 3.93	33	23.9	2.44	1.19, 4.97	36	26.9	1.99	0.96, 4.12
					*p* trend = 0.033				*p* trend = 0.011				*p* trend = 0.025	
**BMI**														
≤23.15	84	22.0	110	40.4	1.0	Referent	54	39.1	1.0	Referent	56	41.8	1.0	Referent
23.16–24.97	121	31.7	71	26.1	0.92	0.52, 1.63	33	23.9	1.47	0.76, 2.84	38	28.4	0.46	0.18, 1.15
24.98–27.40	128	33.5	65	23.9	0.79	0.44, 1.39	32	23.2	1.34	0.69, 2.60	33	24.6	0.35	0.14, 0.87
>27.40	49	12.8	26	9.6	0.42	0.24, 0.75	19	13.8	0.60	0.32, 1.14	7	5.2	0.24	0.10, 0.59
					*p* trend = 0.001				*p* trend = 0.004				*p* trend = 0.004	
**WEIGHT (Kg)**														
≤72	94	24.6	102	37.5	1.0	Referent	52	37.6	1.0	Referent	50	37.3	1.0	Referent
73–80	157	41.0	93	34.1	0.56	0.30, 1.03	42	30.4	0.81	0.39, 1.66	51	38.0	0.33	0.13, 0.82
81–87	78	20.4	51	18.7	0.56	0.31, 1.00	27	19.5	0.97	0.48, 1.96	24	17.9	0.27	0.11, 0.64
>87	53	13.8	26	9.5	0.27	0.13, 0.52	17	12.3	0.44	0.20, 0.96	9	6.7	0.14	0.05, 0.38
					*p* trend = 0.001				*p* trend = 0.021				*p* trend = 0.001	

OR adjusted for all continuous variables in the table; significance of p value for trend < 0.05.

Overall, participants in the highest quartile of height were more likely to be diagnosed with *testicular* germ cell tumors (TGCTs) than participants in the lowest quartile of height, OR 2.22 (95% confidence intervals (CI): 1.25–3.93; adjusted; *p*_trend_ = 0.033). Moreover, histological seminoma subgroup was significantly associated with tallness, very tall men (>182 cm) having a seminoma TGCT risk of OR = 2.44 (95% confidence intervals (CI): 1.19–4.97; adjusted; *p*_trend_ = 0.011). The multivariate-adjusted odds ratio for the highest quartile group of BMI relative to the lowest was 0.42 (95% CI: 0.24–0.75; *p*_trend_ = 0.01), with no difference by histological subtype (Table 2). There was also a significant inverse association of TGCT with increasing weight (*p*_trend_ = 0.001; age-adjusted analysis) and this association was equally present in both histological subgroups.

## Discussion

These preliminary results indicate that TC is positively associated with height, in particular with seminoma subtype, and inversely related with BMI. Therefore, the present study indicated an excess of tall men in the cohort of patients with TC. To our knowledge, this study is the first to explore the relationship between tallness and the risk of TC from a southern European country. Thus far, the majority of studies on tallness in relation to the risk of TC were conducted in US and Nordic countries (Akre et al., 2000; McGlynn et al., 2007; Dieckmann et al., 2008; Cook et al., 2010; Lerro et al., 2010). Measures of adult height, as well as the incidence rate of TC, resulted both elevated among Scandinavian populations (Richiardi et al., 2003; Purdue et al., 2005). Though differences in genetic backgrounds among Scandinavian and southern European populations exist, these preliminary findings imply that possible postnatal exposures may play an important role in determining risk of TC. As adult height is largely determined during the first 2 years of life, it may be postulated that high calorie nutrition after birth could have a role in TC pathogenesis (Frankel et al., 1998; McGlynn et al., 2007; Giannandrea, 2009). In addition, as final adult height is strongly dependent on testis sex-steroids (Veldhuis et al., 2005) a correlation between height and TC risk may suggest that androgen secretion during puberty might be involved in TC progression. One further height-related factor may be insulin-like growth factor (IGF) and the IGF pathway, which is also involved in the control of spermatogenesis (Gunnell et al., 2001; Giovannucci et al., 2004). Increased serum IGF-1 levels were associated with increased height has also been reported in several studies (Chia et al., 2008).

Height has been positively associated with risk of TC in the majority of studies in which it has been examined (Akre et al., 2000; McGlynn et al., 2007; Dieckmann et al., 2008; Cook et al., 2010; Lerro et al., 2010). Akre et al. (2000) found a statistically significant increased risk of TC associated with greater height, and this association was particularly evident in men with seminomas. Dieckmann et al. (2008) found that very tall men (>195 cm) carried a TC risk of OR: 3.35 (95% confidence intervals (CI): 2.88–3.90; adjusted). In the STEED Study, increased height was significantly related to risk of TC (OR: 1.85; CI 95%: 0.71, 1.32) (McGlynn et al., 2007; Lerro et al., 2010).

A number of studies have so far reported an inverse relationship between BMI and TGCTs, although the available results are still conflicting, with some studies reporting null findings (Petridou et al., 1997; Bjørge et al., 2006; Lerro et al., 2010). A recent meta-analysis conducted by Lerro et al. (2010) has produced a tentative evidence that TC risk was inversely associated with BMI, with a summary OR of 0.92 (95% CI: 0.86–0.98; *p* = 0.011). The biological reasons of this negative association are likely to be more complex of those contemplated to explain the association of TC with tallness. Some authors have suggested that lower BMI found in previous studies could also be a mathematical artifact as this association may be related to an excess of tall men in TC cohorts rather than to leanness of the patients (Dieckmann and Pichlmeier, 2002). This is due to the fact that height is squared in the denominator of the BMI-formula, lowering the numerical calculation of MBI in very tall men at hight TC risk despite normal weight. However, our study shows additional significant inverse association of TGCT with increasing weight (*p*_trend_ = 0.001), therefore confuting, at least in part, this suggestion.

Body size is primarily determined by hereditary, nutritional, and hormonal factors (Dieckmann and Pichlmeier, 2002). The hormonal determinants can be divided into growth hormones and sex hormones. Most previous studies on postnatal risk factors for TC have focused on endogenous sex hormones. Obesity is inversely associated with both total testosterone and sex hormone-binding globulin (Akre et al., 2000). Furthermore, obese men have increased levels of both estradiol and estrone coming mostly from extraglandular conversion of androgen precursors (Akre et al., 2000; Dieckmann and Pichlmeier, 2002).

Since testicular germ cell cancer is probably initiated *in utero*, postnatal hormones are likely to function as promoters. These hormones may continue to exert their effect in advanced stages of tumor development at time of puberty when the testicles grow and develop rapidly.

Adult stature can be considered a proxy of childhood nutrition, although stature is also determined by genetic and hormonal factors. It has been suggested that the trend of increasing adult height and the increasing TC incidence are biologically interconnected with improved nutrition in early life.

In summary, we have found a lower risk of TC among men with high BMI. Further investigation of this inverse relationship may be warranted, for which the present results provide only limited support. Furthermore, tallness was positively associated with risk of TC. The biologic mechanism suggested is the promotion of this cancer by sex hormones and/or growth hormones, such as growth hormone and/or IGF-I.

## Funding

Financial support for this work was provided by a Grant from the Italian Ministry of Education and Research (MIUR-PRIN) and the University of Rome “La Sapienza” Faculty of Medicine.

### Conflict of interest statement

The authors declare that the research was conducted in the absence of any commercial or financial relationships that could be construed as a potential conflict of interest.
